# Sudden extensive bloody pleural and pericardial effusion in a subject with untreated known hypothyroidism after total thyroidectomy, triggered by pneumonia

**DOI:** 10.1186/s12902-022-01146-9

**Published:** 2022-09-17

**Authors:** Yuichiro Iwamoto, Fuminori Tatsumi, Yukino Katakura, Kazunori Dan, Ryo Wamata, Tomohiko Kimura, Masashi Shimoda, Shuhei Nakanishi, Kohei Kaku, Tomoatsu Mune, Hideaki Kaneto

**Affiliations:** Department of Diabetes, Endocrinology and Metabolism, Kawasaki Medical School, 577 Matsushima, Kurashiki, 701-0192 Japan

**Keywords:** Hypothyroidism, Bloody pleural effusion, Bloody pericardial fluid, Case report

## Abstract

**Background:**

In subjects with hypothyroidism, edema is often observed, and pleural effusion and pericardial fluid could be also observed. The color of such fluid retention is usually yellow. Here we show a very rare case with hypothyroidism who had bloody pleural effusion and bloody pericardial fluid.

**Case presentation:**

A 42-year-old male noticed chest pain and the aggravation of exertional dyspnea, and he was transported to our institution by emergency. He had Graves’ disease and underwent total thyroidectomy about 4 years before. After then, he had been treated with 200 μg/day of levothyroxine sodium for the maintenance of thyroid function. However, he self-interrupted such medication about 2 years before. Thyroid function on admission was reduced as follows: free triiodothyronine, 1.60 pg/mL; free thyroxine < 0.40 ng/dL; thyroid-stimulating hormone 25.50 μU/mL. Inflammation markers were increased: white blood cells 25,280 /μL; C-reactive protein 18.66 mg/dL. A large amount of pericardial fluid and pleural effusion were observed in chest and abdominal computer tomography and echocardiography. In addition, we performed pleural effusion and pericardial fluid collection. Pleural effusion in this subject showed bloody color, but not yellow. In cell block specimen of pleural effusion and pericardial fluid, red blood cells, neutrophils and lymphocyte component were observed. In this subject, however, we were unable to find any obvious background disease causing bloody pericardial effusion. Finally, we concluded that bloody pleural effusion and bloody pericardial fluid were brought about in a subject with untreated known hypothyroidism after total thyroidectomy, triggered by pneumonia.

**Conclusions:**

In subjects with hypothyroidism, fluid and mucopolysaccharide are stored in interstitial space and protein osmolality is increased, thus leading to edema and fluid retention. It is noted here that pleural effusion and pericardial fluid in this subject showed bloody color and included red blood cells. There are no reports of bloody pericardial fluid with hypothyroidism. Therefore, it is important to keep in mind that a subject with some trigger, such as infection, may have a hematologic fluid retention that is not seen when hypothyroidism is present alone, as observed in this subject.

## Background

Graves’ disease is hyperthyroidism which is characterized by diffuse goiter, exophthalmos and palpitation. When a total thyroidectomy is performed for Graves’ disease, thyroid hormone preparations such as levothyroxine sodium are commonly administered to maintain thyroid function. Chronic lack of thyroid hormone replacement therapy after total thyroidectomy increases the risk of mood disorders, cardiovascular disorders, and osteoporosis [1]. In addition, in subjects with hypothyroidism, edema is often observed and pleural effusion and pericardial fluid could be also observed. In hypothyroidism, fluid retention occurs along with mucopolysaccharides in the interstitium, resulting in edema and fluid retention. Fluid accumulation with mucopolysaccharides is often yellow in color because of the abundance of protein, and rarely bloody because of the absence of red blood cell components in the accumulation [2, 3]. In general, bloody pleural effusion is observed in subjects with malignancy, tuberculous pleural inflammation, pulmonary thrombosis and external wound [4].

Here, we present a patient who presented with sudden bloody pericardial effusion and bloody pleural effusion simultaneously with pneumonia in a background of hypothyroidism due to prolonged interruption of thyroid hormone preparations.

## Case presentation

A 42-year-old male noticed chest pain and the aggravation of exertional dyspnea, and he was transported to our institution by emergency. He had Graves’ disease accompanied by exophthalmos and palpitation and underwent total thyroidectomy about 4 years before. After then, he had been treated with 200 μg/day of levothyroxine sodium for the maintenance of thyroid function. However, he self-interrupted such medication about 2 years before. He somtimes had felt exertional dispnea since 1 year before, and he finally noticed chest pain and the aggravation of exertional dyspnea about 2 weeks before. There was no family history of thyroid disease or heart disease. He had a smoking history with a Brinkman index of 440. He had no history of alcohol consumption.

**Fig. 1:**
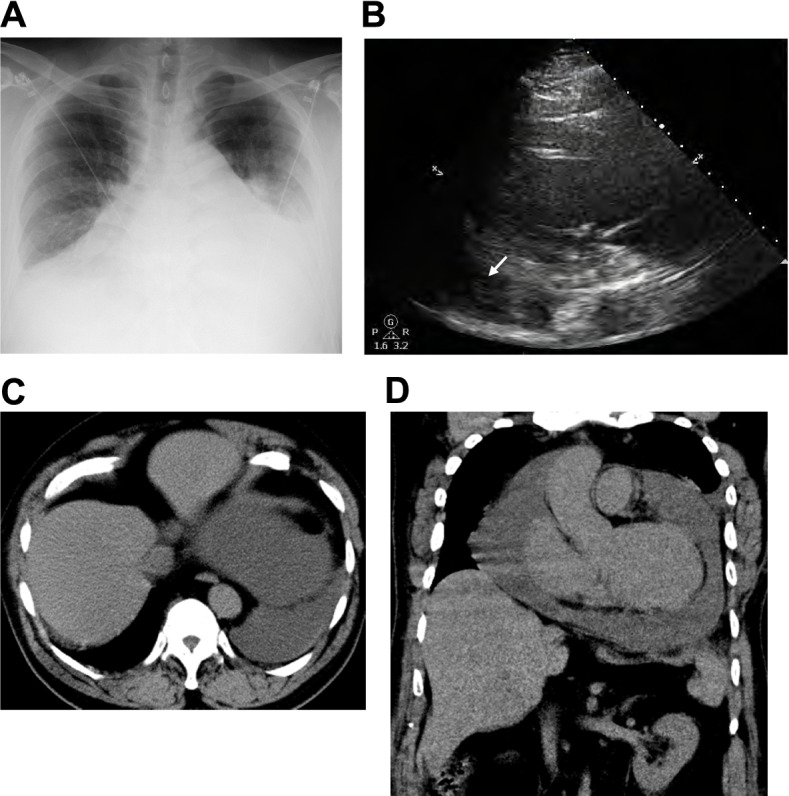
**A** Pleural effusion was observed in chest X-ray. **B** A large amount of pericardial fluid was observed in echocardiography (arrow). **C**, **D** A large amount of pleural effusion and pericardial fluid were observed in chest and abdominal computer tomography (CT)

On admission, his height, body weight and body mass index were 180 cm, 118 kg and 35.2 kg/m^2^, respectively. Blood pressure, heart rate and body temperature were 155/103 mmHg, 79 /min and 36.3 °C. Saturation of percutaneous oxygen (SpO_2_) was 78% at room air. Respiratory sounds were attenuated in bilateral lung fields. Heart sounds were weak, and no pericardial friction sounds were heard. There was no edema in the extremity, and there were no other physical findings of note. Table 1 shows the data on admission in this subject. The thyroid-stimulating hormone (TSH) was high and free triiodothyronine (FT_3_) and free thyroxine (FT_4_) were low, indicating hypothyroidism after total thyroidectomy. White blood cell (WBC) and C-reactive protein (CRP) levels were also increased. Increased liver enzymes, renal dysfunction and hyperglycemia were also present. In addition, pleural effusion was observed in chest X-ray (Fig. 1A). A large amount of pericardial fluid was observed in transthoracic echocardiography (Fig. 1B, arrow), although the ejection fraction (EF) was about 60%. A large amount of pericardial fluid and pleural effusion was observed in chest and abdominal computer tomography (CT) (Fig. 1C). In addition, there was a consolidation in the left lower lobe that was suspicious of pneumonia and a pressure-drain atelectasis due to pleural and pericardial effusions. Thyroid ultrasound was performed but no residual thyroid tissue was found. Based on these findings, we diagnosed him with hypothyroidism and subsequent fluid retention due to self-interruption of thyroid hormone replacement therapy with levothyroxine sodium. In addition, there was emphysematous change in the left lower lobe. The patient was treated for hypoxemia with nasal high flow and finally SpO_2_ was increased up to 100%.

**Table 1:** Clinical data on admission in this subject

Peripheral blood		Diabetes markers		Electrolytes	
Red blood cells	497 × 10^4^ /μL	Plasma glucose	247 mg/dL	Sodium	137 mEq/L
Hemoglobin	15.5 g/dL	HbA1c	6.4%	Potassium	3.2 mEq/L
White blood cells	25,280 /μL			Chloride	97 mEq/L
Neutrophils	67.0%	Lipid markers		Calcium	6.9 mg/dL
Eosinophils	0.0%	LDL-cholesterol	138 mg/dL	Phosphorous	8.7 mg/dL
Lymphocytes	15.0%	HDL-cholesterol	28 mg/dL	Magnesium	2.3 mg/dL
Platelet	46.3 × 10^4^ /μL	Triglyceride	104 mg/dL		
Blood biochemistry		Endocrine markers		Inflammation marker	
Total protein	7.9 g/dL	TSH	25.50 μU/mL	CRP	18.66 mg/dL
Albumin	3.6 g/dL	FT3	1.60 pg/mL	Infection	
Total bilirubin	2.1 mg/dL	FT4	< 0.40 ng/dL	Adenovirus	< 4-fold
AST	90 U/L	TSAb	498%	Coxsackievirus	< 4-fold
ALT	71 U/L	TRAb	16.6%	Echovirus	< 4-fold
γ-GTP	134 U/L	Intact-PTH	41 pg/mL	Influenza virus	< 10-fold
LDH	488 U/L	Immune markers		C7-HRP	Negative
Creatinine	1.61 mg/dL	Anti-nuclear Ab	40-fold	T-SPOT	Negative
BUN	19 mg/dL	Rheumatoid factor	< 15 U/mL	HIV	Negative
CK	888 U/L	PR3-ANCA	< 1.0 U/mL		
CK-MB	11 U/L	MPO-ANCA	< 1.0 U/mL		

*Abbreviation: AST* aspartate aminotransferase, *ALT* alanine aminotransferase, *γ-GTP* γ-glutamyl transpeptidase, *LDH* lactate dehydrogenase, *BUN* blood urea nitrogen, *CK* creatine kinase, *HbA1c* hemoglobin A1c, *LDL* low density lipoprotein, *HDL* high density lipoprotein, *TSH* thyroid-stimulating hormone, *FT3* free triiodothyronine, *FT4* free thyroxine, *TSAb* thyroid-stimulating antibody, *TRAb* thyrotropin receptor antibody, *PTH* parathyroid hormone, *Ab* antibody, *PR3-ANCA* proteinase 3 antineutrophil cytoplasmic antibody-associated vasculitis, *MPO-ANCA* myeloperoxidase antineutrophil cytoplasmic antibody-associated vasculitis, *CRP* C-reactive protein, *HIV* human immunodeficiency virus

**Fig. 2:**
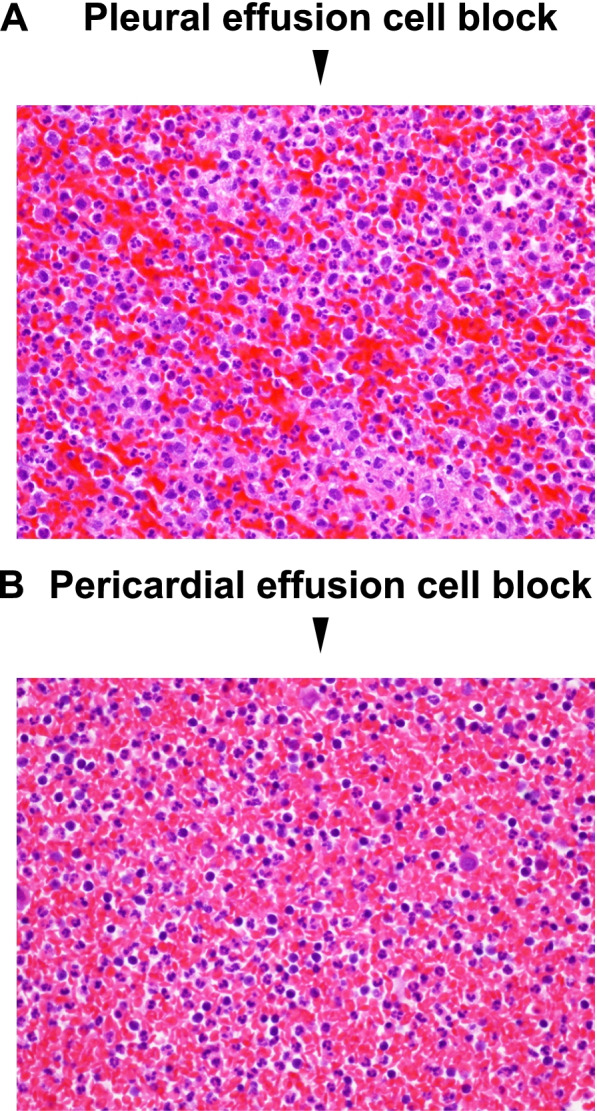
**A** In cell block specimen of pleural effusion, red blood cells, neutrophils and lymphocyte component were observed. **B** In cell block specimen of pericardial fluid, red blood cells, neutrophils and lymphocyte component were similarly observed

In addition, we performed pleural effusion collection. Pleural effusion in this subject showed bloody color, but not yellow. As shown in Fig. 2A, in cell block specimen of pleural effusion, red blood cells, neutrophils and lymphocyte component were observed. There were no malignancy findings in pleural effusion. The characteristics of pleural effusion are as follows: pH, 7.8; specific gravity, 1.035; total protein, 5.2 g/dL; glucose, 120 mg/dL; adenosine deaminase (ADA), 18.8 U/L; LDH, 292 U/L; amylase, 19 U/L; carcinoembryonic antigen (CEA), 3.2 ng/mL; carbohydrate antigen 19–9 (CA19–9), < 5.0 U/mL. We thought that characteristics of pleural effusion in this subject was exudative, but not transudative, based on Light’s criteria. Furthermore, we performed pericardial fluid collection. Pericardial fluid also showed bloody color, but not yellow. Cell block specimen of pericardial fluid showed erythrocyte, neutrophil, and lymphocyte components, but no evidence of malignancy. Culture results showed α-streptococcus in sputum culture. Blood, urine, pleural fluid, and pericardial fluid cultures were negative.

Further investigations were performed to determine the cause of the pericardial fluid. The results of the blood tests are listed in Table 1. Pericarditis due to viral pericarditis, tuberculous pericarditis and autoimmune diseases were excluded from the blood test results. The possibility of acute myocardial infarction was ruled out as the creatine kinase-MB (CK-MB) was within normal limits and the patient was asymptomatic on echocardiography after recovery. The WBC was elevated on admission, but we thought that this was due to pneumonia rather than hematological disease.

Regarding the treatment for hypothyroidism, we started 50 μg/day of levothyroxine sodium and gradually increased its dose up to 200 μg/day. In addition, since pneumonia was detected in the lower left lung and inflammation markers such as CRP and WBC were increased, we started antibiotics sulbactam/ampicillin and continued it for 7 days. Two days after admission, pericardial drainage was performed, and a total of drainage was as large as 950 mL. After then, dyspnea was substantially reduced. Therefore, 3 days later, pericardial drainage was removed. Eighteen days later, pericardial fluid and pleural effusion disappeared almost completely in chest and abdominal CT. About 1 month later, thyroid function was normalized. After then, there was no recurrence of pericardial fluid and pleural effusion. Finally, based on the time course of this case in which the recurrence of fluid retention disappeared after thyroid hormone replacement therapy, we concluded that bloody pleural effusion and bloody pericardial fluid were brought about by hypothyroidism in this subject.

## Discussion and conclusions

In this case report, we showed a subject with hypothyroidism with bloody pericardial effusion and bloody pleural effusion, as well as pneumonia, after thyroid hormone replacement therapy had been interrupted for 2 years. The patient developed pneumonia after self-interruption of the thyroid hormone levothyroxine sodium, which was complicated by dyspnea and fluid retention, and such symptoms and fluid retention were relieved after initiation of levothyroxine sodium and antimicrobial agentes. Therefore, we thought that dyspnea and fluid retention were induced by hypothyroidism. It is noted here that his pleural effusion and pericardial fluid were both bloody color, but not yellow, and in cell block specimen of a pleural effusion and pericardial fluid, red blood cells were observed. To the best of our knowledge, there are no reported cases of bloody pleural or pericardial effusions caused solely by hypothyroidism. Therefore, we think that hypothyroidism and another trigger may have caused the patient to present with bloody pleural and pericardial effusions.

**Fig. 3:**
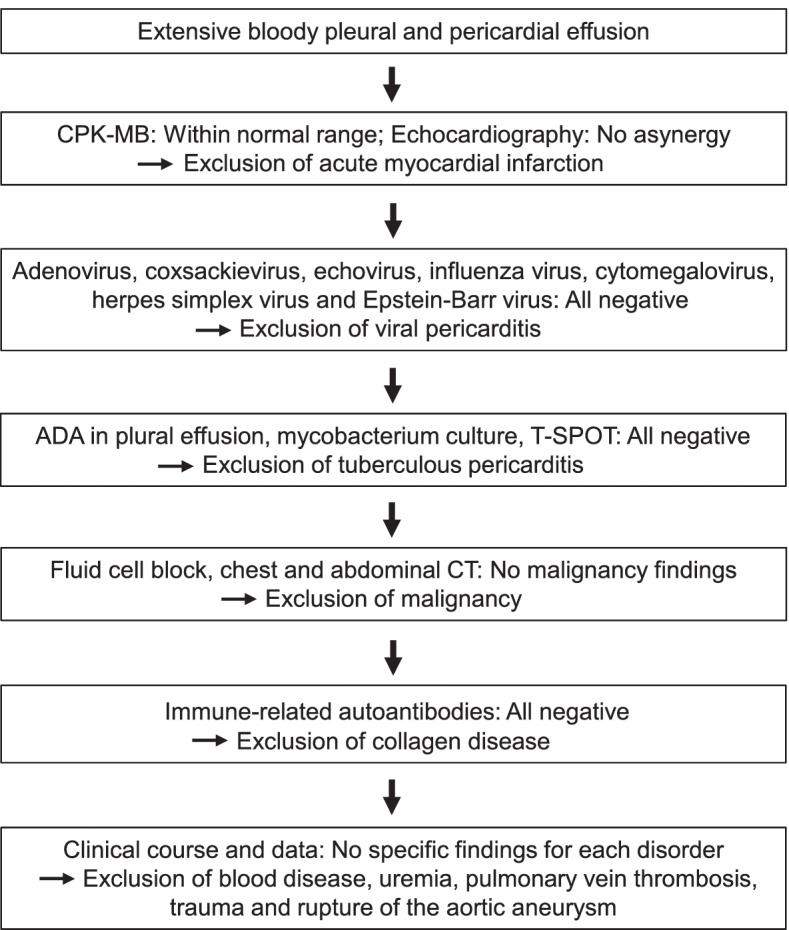
A flowchart regarding the differential diagnosis of bloody pleural and pericardial effusion

Thyroid hormones are involved in metabolism, regulation of the sympathetic nervous system, and growth during childhood, and deficiency of thyroid hormones disrupts the metabolic mechanisms of the body. In subjects with hypothyroidism, fluid and mucopolysaccharide are stored in interstitial space and protein osmolality is increased, thus leading to edema and fluid retention. In addition, the characteristics of pleural effusion in this subject was exudative, which point is compatible with previous reports showing that characteristics of pleural effusion due to hypothyroidism is exudative, but not transudative [2, 3]. It is noted here that pleural effusion and pericardial fluid in this subject showed bloody color and included red blood cells. There is no report that pericardial fluid due to hypothyroidism shows bloody color. A flowchart of the diagnosis of a bloody pericardial fluid and bloody pleural effusion in this case is shown in Fig. 3. Bloody pericardial fluid could be brought about by several diseases such as viral pericarditis, tuberculous pericarditis, acute myocardial infarction, malignancy, collagen disease, blood disease, uremia, pulmonary vein thrombosis, trauma and rupture of the aortic aneurysm [4]. In this subject, however, we excluded the possibility of having these diseases as described in case presentation section. Diseases that can bring about grossly bloody pleural effusion include malignancy, tuberculous pleurisy, pulmonary thromboembolism, and traumatic hemothorax, all of which were negative in this case [4]. Although bacterial pleurisy and pyothorax can also cause bloody pleural effusion, pleural fluid culture test was negative and pleural fluid glucose level was not decreased (120 mg/dL) in this case, which was not compatible with bacterial pleurisy or pyothorax. In addition, there was no report of bloody pleural effusion due to pneumonia-associated pleural effusion. In the case of bloody pleural effusions, malignancy and tuberculosis are more frequent [5], and these tests are prioritized in clinical practice. Other diseases presenting with bloody pleural effusions are less frequent and may delay testing and diagnosis. On the other hand, measurement of thyroid hormones is quite straightforward. If the bloody pleural effusion is due to hypothyroidism, it can be quickly corrected with thyroid hormone replacement. We believe that many clinicians should be aware of this fact.

Hypothyroidism causes increased capillary permeability and impaired lymphatic drainage [6]. There have been case reports of bloody pericardial effusions due to systemic lupus erythematosus (SLE) [7]. Pericardial effusions due to SLE are also usually clear and yellow, but prolonged inflammation and infiltration of the pericardium with large numbers of polymorphonuclear leukocytes and monocytes may result in a bloody pericardial effusion. As numerous inflammatory cells were also detected in this subject’s pericardial and pleural fluid cell block, this subject may have presented with a bloody pericardial and pleural effusion by the same mechanism. Therefore, we concluded that bloody pleural effusion and bloody pericardial fluid were brought about in a subject with untreated known hypothyroidism after total thyroidectomy, triggered by pneumonia.

Fluid retention in hypothyroidism is characterized by a predominance of fluid retention in the upper body, such as pleural effusion and pericardial fluid. It was reported that about 30% of subjects with hypothyroidism had pericardial fluid and that most of them are reduced by thyroid hormone replacement therapy [8]. In this subject, however, a total of pericardial drainage was as large as 950 mL which was much larger than that in the previous reports. In addition, it was reported that cardiac tamponade due to hypothyroid was recovered within a few days by pericardial drainage and thyroid hormone replacement therapy [9]. Therefore, it is important to differentiate pericardial effusion due to hypothyroidism as early as possible in cases of unexplained pericardial effusion. We think that starting levothyroxine sodium soon after admission could help to prevent the recurrence of pericardial fluid in this subject. In addition, discontinuation of thyroid hormone replacement therapy in patients with documented hypothyroidism should be avoided as it can lead to serious complications such as pleural and/or pericardial effusions.

Severe hypothyroidism causes hypothermia and bradycardia. It is known that in severe conditions with a marked inflammatory response as observed in this case, an adaptive response to reduced thyroid hormone demand can reduce catabolism to protect the body [10]. In hypothyroidism, bradycardia may not be present even in cases where bradycardia should be present such as in cardiac tamponade [11]. It is assumed that a similar mechanism is responsible for the reduced tachycardic response in the present case. In most cases of serious diseases, there are other important signs besides vital signs, but in the case of hypothyroidism, vital signs may not be serious at first glance as observed in this case, and thus we must be careful about this point.

Although bloody fluid retention due to hypothyroidism is very rare and its pathogenesis is not known, we experienced a case of hypothyroidism with bloody pleural effusion and bloody pericardial effusion both of which were improved by pericardial drainage and thyroid hormone replacement therapy after excluding the possibility of other diseases. Taken together, we must bear in mind that bloody pleural effusion and bloody pericardial effusion that is not seen when hypothyroidism is present alone can be brought on by hypothyroidism when complicated by other serious diseases such as infection.

## Data Availability

Not applicable.

## Abbreviations

FT3: Free triiodothyronine
FT4: Free thyroxine
TSH: Thyroid-stimulating hormone
WBC: White blood cells
CRP: C-reactive protein
CT: Computer tomography
SpO_2_: Saturation of percutaneous oxygen
AST: Aspartate transaminase
ALT: Alanine aminotransferase
γ-GTP: γ-glutamyl transpeptidase
BUN: Blood urea nitrogen
EF: Ejection fraction
ADA: Adenosine deaminase
CEA: Carcinoembryonic antigen
CA 19–9: Carbohydrate antigen 19–9
SLE: Systemic lupus erythematosus
